# Supplementary material for: Sex Plays a Multifaceted Role in Asthma Pathogenesis
Source: Biomolecules. 2022 Apr 29;12(5):650. doi: 10.3390/biom12050650 (PMC9138801; doi:10.3390/biom12050650)
Supplement: Supplementary file 1 [file biomolecules-12-00650-s001.zip › biomolecules-1656925-supplementary.pdf]

biomolecules

Sex plays a multifaceted role in asthma pathogenesis

Tomomitsu Miyasaka , Kaori Dobashi-Okuyama, Kaori Kawakami, Chiaki Masuda-Suzuki, Motoaki Takayanagi and Isao Ohno

Detailed methodology for Figure 1: (A) C57BL/6 wild-type mice (age, 6–8-weeks) were sensitized via intraperitoneal injection of 8 μg OVA (Grade V; Sigma–Aldrich, St Louis, MO, USA) adsorbed with 4 mg aluminum hydroxide (Wako Pure Chemical Industries, Osaka, Japan) in 500 μL saline on days 0 and 5. On day 26, mice were challenged with aerosolized OVA (0.5% in saline) for 1 h twice, 4 h apart. Mice were euthanized at 1, 4, and 16 h after OVA inhalation. Ep-CAM$^+$ airway epithelial cells in the lungs that were stained with PE anti-mouse CD326 (Ep-CAM) antibody (Clone G8.8; BioLegend, San Diego, CA, USA), were isolated using an autoMACS Separator (Miltenyi Biotec, Bergisch Gladbach, Germany) and Anti-PE MicroBeads UltraPure (Miltenyi Biotec). Total RNA was extracted from Ep-CAM$^+$cells, using a ReliaPrep RNA cell miniprep system (Promega Corporation, Madison, WI, USA). First-strand cDNA was synthesized using the PrimeScript RT reagent kit (TaKaRa Bio, Otsu, Japan), according to the manufacturer's instructions. RT-PCR was performed using gene-specific primers, the Power SYBR Green PCR Master Mix (Applied Biosystems, Foster City, CA, USA), and a StepOnePlus Real-Time PCR system (Applied Biosystems). The primer sequences used for amplification were as follows: *Il33*, forward 5′-TAACACAGTCTCCTGCCTCCCT-3′; *Il33*, reverse 5′-TGGTCTTTTCCAGAGTCGTCAACA-3′; *Ccl2*, forward 5′-TTTCCACAACCACCTCAAGCAC-3′; *Ccl2,* reverse 5′-TAAGGCATCACAGTCCGAGTCA-3′; *Hprt*, forward 5′-TTGGGCTTACCTCACTGCTTTCC-3′; *Hprt*, 5′-reverse ATCATCGCTAATCACGACGCTGG-3′. The expression levels of the target genes and *Hprt* as a reference gene were calculated for each

sample using the reaction efficiency, as determined by performing amplifications using standards. (B) Human epithelial cell line BEAS-2B was incubated in Dulbecco's modified Eagle medium/F-12, no phenol red medium (Thermo Fisher Scientific, Waltham, MA, USA) with Small Airway Epithelial Growth Medium Supplements (Lonza, Basel, Switzerland) without hydrocortisone in the presence or absence of 17β-estradiol (1×10^{-7} M; Sigma-Aldrich) for 15 d. BEAS-2B cells were stimulated with 50 ng/mL TNF-α (PeproTech, Rocky Hill, NJ, USA) in the presence or absence of 17β-estradiol for 2 d. On day 17, the supernatants were stored at −80 °C. CCL2 concentration was assayed using enzyme-linked immunosorbent assay kits (eBioscience, San Diego, CA, USA). The detection limit was 7 pg/mL. (C) Human $CD14^+$ cells from the peripheral blood of a single healthy volunteer were incubated with the supernatants collected in experiment B. Three days after incubation, the proportion of $CD86^+$ $CD1c^+$ cells among $CD14^+$ cells were determined via flow cytometry. Cells (1×10^6 cells/100 μL) were pre-incubated with anti-FcγRII and III mAb (Clone 93; BioLegend) on ice for 15 min in PBS containing 1% FCS and 0.1% sodium azide. To identify activated and differentiated DCs among monocytes, the cells were stained with PE-conjugated anti-CD86 (Clone IT2.2; BioLegend), PerCP-conjugated anti-CD14 (Clone M5E2; BioLegend), and PE/Cy7-conjugated anti-CD1c (Clone L161; BioLegend). Dead cells stained using the LIVE/DEAD fixable blue dead cell stain kit (Thermo Fisher Scientific) were excluded from analysis. Data from these experiments were analyzed using Diva (BD Biosciences, San Jose, CA) software. (D) BAL fluids were prepared 2 d after OVA or saline inhalation with 2 × 0.25 mL chilled PBS. After centrifugation at 450 × g for 10 min at 4 ℃, the supernatants were stored at −80 ℃ for the culture experiments. $CD11c^+$ cells were isolated from the spleen using an autoMACS Separator (Miltenyi Biotec) and Anti-CD11c MicroBeads (Miltenyi Biotec). $CD11c^+$ cells were cultured in mixed RPMI

1640 complete medium supplemented with 10% fetal calf serum and 2-mercaptoethanol with the pooled BAL fluids at a ratio of 1:1. The expression levels of *Cd86* mRNA in the cultured CD11c$^+$ cells were analyzed 48 h after culturing via RT-PCR as described in experiment A. The primer sequences used for amplification were: *Cd86*, forward 5′-GCCCAGCAACACAGCCTCTAA-3′; *Cd86*, reverse 5′-GCTCTCACTGCCTTCACTCTGC-3′.
